# Elusive Copy Number Variation in the Mouse Genome

**DOI:** 10.1371/journal.pone.0012839

**Published:** 2010-09-21

**Authors:** Avigail Agam, Binnaz Yalcin, Amarjit Bhomra, Matthew Cubin, Caleb Webber, Christopher Holmes, Jonathan Flint, Richard Mott

**Affiliations:** 1 Wellcome Trust Centre For Human Genetics, Oxford, United Kingdom; 2 Department of Physiology, Anatomy and Genetics, Henry Wellcome Building for Gene Function, Oxford, United Kingdom; 3 Department of Statistics, Henry Wellcome Building for Gene Function, Oxford, United Kingdom; University of California, United States of America

## Abstract

**Background:**

Array comparative genomic hybridization (aCGH) to detect copy number variants (CNVs) in mammalian genomes has led to a growing awareness of the potential importance of this category of sequence variation as a cause of phenotypic variation. Yet there are large discrepancies between studies, so that the extent of the genome affected by CNVs is unknown. We combined molecular and aCGH analyses of CNVs in inbred mouse strains to investigate this question.

**Principal Findings:**

Using a 2.1 million probe array we identified 1,477 deletions and 499 gains in 7 inbred mouse strains. Molecular characterization indicated that approximately one third of the CNVs detected by the array were false positives and we estimate the false negative rate to be more than 50%. We show that low concordance between studies is largely due to the molecular nature of CNVs, many of which consist of a series of smaller deletions and gains interspersed by regions where the DNA copy number is normal.

**Conclusions:**

Our results indicate that CNVs detected by arrays may be the coincidental co-localization of smaller CNVs, whose presence is more likely to perturb an aCGH hybridization profile than the effect of an isolated, small, copy number alteration. Our findings help explain the hitherto unexplored discrepancies between array-based studies of copy number variation in the mouse genome.

## Introduction

Array comparative genomic hybridization (aCGH) using long oligonucleotides (>50 bp) has emerged as a preferred technology for genome-wide detection of copy number variation, structural variation in DNA greater than 1 kilobase in size. ACGH experiments have already shown that more than 3% of the human genome is affected by copy number variants (CNVs) [1], [2], that there is a relationship between expression variation and copy number variation [3], [4], [5], [6], and that CNVs contribute to disease susceptibility [1], [7], [8], [9].

However the inadequacies of aCGH are also widely acknowledged [10]. First, estimates of the amount of copy number variation differ considerably: for example the fraction of the mouse genome estimated to be copy number variant ranges from 3% [6] to 10.7% [5]. Second, concordance between CNVs from differing aCGH experiments is low: in human studies discrepancies occur between analyses that assessed identical samples [2], [11], [12], [13]; in mouse studies there is low overlap between CNVs reported for the same inbred strains (37% between [14] and [5]).

A number of explanations for the inconsistent results between aCGH experiments have been considered [10]. These include the use of different arrays and CNV detection algorithms, with varying sensitivity, specificity and probe density [5], [10], as well as technical problems with aCGH arising from, for example, the interference of SNPs with hybridization [15]. However the relative contribution of each factor to the low concordance between studies is not known.

We set out to quantify the factors that affect the reproducibility of aCGH studies. To do this we compared four published mouse long-oligonucleotide aCGH experiments with our own analysis of CNVs in new aCGH data from seven inbred strains of mice (*A/J*, *AKR/J*, *BALB/cJ*, *C3H/HeJ*, *CBA/J*, *DBA/2J* and *LP/J*) using a 2.1 million probe NimbleGen array. The use of inbred mice, in conjunction with one common strain used as the reference in all of the studies (*C57BL/6J*), enabled us to focus on the issues associated with the platform and detection algorithms alone, without considering the additional population variability presented in human copy number variation studies. We note here that inaccuracies in the mouse reference assembly will distort detection of CNVs [16], but since this factor will be consistent across genome-wide studies we do not consider its impact further. We followed up our aCGH study with an extensive validation strategy for CNVs that combined PCR, real-time PCR, sequencing, fluorescence *in situ* hybridization to interphase nuclei (FISH) and multiplex ligation-dependent probe amplification (MLPA) [17], in addition to a very high-density array. Our work reveals a complex architecture in mouse CNVs that will make it even harder to validate and compare aCGH experiments than previously thought.

## Results

### Detecting CNVs in Seven Inbred Strains

Using a 2.1 million probe NimbleGen array (2.1M array) we performed CGH experiments for seven inbred mouse strains (*A/J*, *AKR/J*, *BALB/cJ*, *C3H/HeJ*, *CBA/J*, *DBA/2J* and *LP/J*; collectively termed the test strains), all co-hybridized with genomic DNA from *C57BL/6J* (the reference strain). We began our analyses by processing the aCGH data to account for SNP effects on probe hybridization, as previous work suggests that this will improve the specificity and sensitivity of automated CNV detection [15].

**Figure 1:**
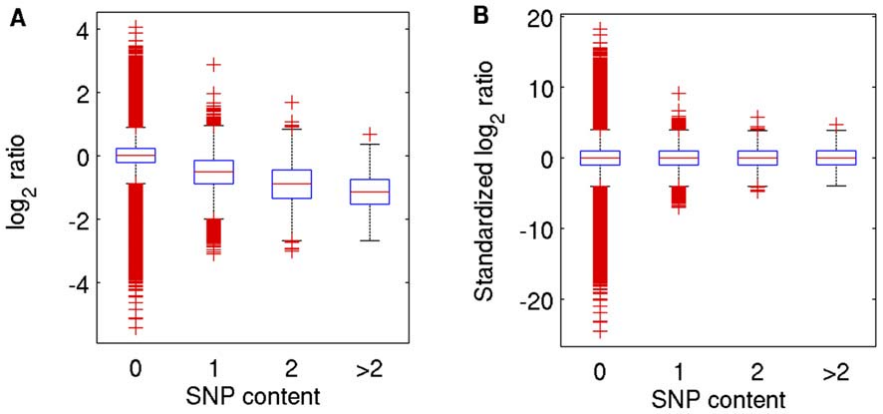
Distribution of log_2_ ratios from probes with and without SNPs, before and after standardization. **A**: Box and whisker plots of log_2_ ratios from probes with zero, one, two and more than two SNPs in their sequence in the *A/J* versus *C57BL/6J* experiment (normal dye); the boxes represent the inter-quartile ranges of the distributions, the whiskers are 1.5 times the inter-quartile range, and red crosses are outliers. The median log_2_ ratios from probes with zero, one, two and three or more SNPs are 0.02, −0.50, −0.88 and −1.14, respectively. **B**: Box and whisker plots of the standardized log_2_ ratios from probes with zero, one, two and more than two SNPs.

**Figure 2:**
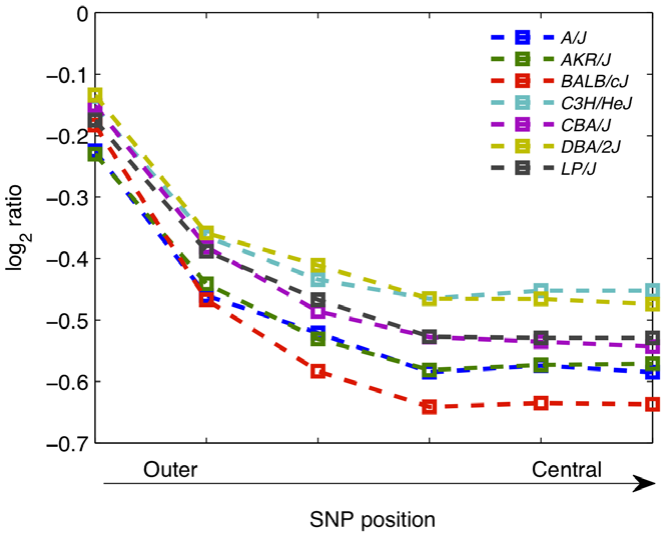
SNP effect depends on position within probe. Log_2_ ratios of probes containing one SNP were classified by the position of the SNP within the probe (probes were divided into 11 segments of equal length, and symmetrical segments combined to form one category; so log_2_ ratios from probes with SNPs in either of the outermost segments were grouped together, and so on). Shown here are the median log_2_ ratios from each category, in each strain.

We quantified the impact of SNPs on hybridization (measured as the log_2_ transformed ratio of the test versus reference hybridization signals). The effect is surprisingly large: by matching probe location to the set of 8.27 million SNPs published by Perlegen Sciences (CA,USA) [18] (Tables S1 and S2), we found that each SNP decreases the log_2_ ratio by 0.5 on average (so adding two SNPs halves the hybridization signal) (Table 1 and Figure 1A); Figure 1B depicts the distributions of log_2_ ratios after they have been standardized for probe SNP content. In addition, the effect of a SNP depends on its position within a probe (Figure 2 and Table S3). This observation is consistent with our analysis of the effects of SNPs on expression quantitative trait loci (eQTLs) [19].

**Table 1:** Probe SNP content versus probe hybridization.

	SNP content				Regression on SNP content		
Strain	0	1	2	>2	*P*-value	slope	R^2^
*A/J*	0.02	−0.50	−0.88	−1.14	0	−0.50	0.040
*AKR/J*	0.02	−0.50	−0.87	−1.18	0	−0.50	0.049
*BALB/cJ*	0.04	−0.54	−1.03	−1.25	0	−0.57	0.054
*C3H/HeJ*	0.03	−0.40	−0.86	−1.23	0	−0.44	0.043
*CBA/J*	0.05	−0.45	−0.84	−1.15	0	−0.46	0.036
*DBA/2J*	0.03	−0.40	−0.86	−1.20	0	−0.44	0.038
*LP/J*	0.02	−0.45	−0.83	−0.94	0	−0.47	0.053
*A/J* (d.s.)	0.03	−0.50	−1.00	−1.24	0	−0.54	0.061
*AKR/J* (d.s.)	0.06	−0.50	−1.03	−1.45	0	−0.57	0.057
*BALB/cJ* (d.s.)	0.04	−0.52	−1.00	−1.30	0	−0.56	0.051
*C3H/HeJ* (d.s.)	0.06	−0.55	−1.13	−1.48	0	−0.61	0.059
*CBA/J* (d.s.)	0.04	−0.53	−1.08	−1.58	0	−0.57	0.051
*DBA/2J* (d.s.)	0.03	−0.61	−1.03	−1.31	0	−0.61	0.060
*LP/J* (d.s.)	0.03	−0.45	−0.80	−1.04	0	−0.48	0.046

For each strain, the median log_2_ ratios for probes with zero, one, two and more than two SNPs are shown. A linear regression analysis was conducted to fit a linear model to the data for each strain. The *P*-value, slope and the square of the correlation coefficient (R) are shown for all the experiments (d.s.  =  dye swap).

We called CNVs using SW-ARRAY [20]. We found 1,477 deletions across the seven test strains, and 499 gains. Table S4 gives the CNV coordinates. Deletions have a median length of 44.2 Kb and cover an average of 33.3 Mb per strain (1.3%), whereas gains have a median length of 53.2 Kb and cover only 13.8 Mb per strain (0.54%) (Table 2). The minimum detectable CNV length was ∼1 Kb, corresponding to the probe spacing on our array (median 1,136 bp), and the use of SW-ARRAY and permutation testing to assess the significance of any CNV call; in practice the majority of CNVs were longer than this (10^th^ percentile 7.9 Kb). For some of our analyses it was useful to merge overlapping CNVs detected in different strains into CNV regions [2], [5], [6]; merging yielded 600 deletion- and 183 gain-CNV regions (Table S5). Of the 600 deletion-CNV regions, 330 are present in more than one strain (55%). 108 out of the 183 gain-CNV regions are present in more than one strain (59%). Combining these two sets gives 755 non-overlapping CNV regions. Approximately 113 Mb (4.4%) of the *C57BL/6J* genome is identified as a CNV region. Fifteen regions contain both deletions and gains. Our estimate of CNV content is comparable to that predicted by a similar study (3% [6]), but much less than that observed in Henrichsen et al. [5] (10.7%).

**Table 2:** Numbers, total sizes and fraction of genome coverage, relative to *C57BL/6J*, of putative deletions and gains found in five long-oligonucleotide aCGH studies of CNV in the mouse genome.

		Deletions			Gains			Abnormal CNVs		
Study	Strain	No.	Mb	%	No.	Mb	%	No.	Mb	%
Agam et al.	*A/J*	183	26.42	1.03	78	12.38	0.48			
"	*AKR/J*	193	35.00	1.36	70	11.64	0.45			
"	*BALB/cJ*	253	38.15	1.48	98	17.20	0.67			
"	*C3H/HeJ*	340	43.35	1.69	83	16.90	0.66			
"	*CBA/J*	181	29.65	1.15	70	8.41	0.33			
"	*DBA/2J*	206	39.99	1.56	44	12.94	0.50			
"	*LP/J*	121	20.35	0.79	56	17.10	0.67			
"	Mean	211	33.27	1.29	71	13.79	0.54			
Graubert et al.	*A/J*	1	0.42	0.02	2	0.53	0.02			
"	*AKR/J*	10	1.59	0.06	4	0.74	0.03			
"	*BALB/cJ*	-	-	-	-	-	-			
"	*C3H/HeJ*	8	1.52	0.06	2	0.53	0.02			
"	*CBA/J*	-	-	-	-	-	-			
"	*DBA/2J*	10	1.08	0.04	2	0.53	0.02			
"	*LP/J*	-	-	-	-	-	-			
"	Mean	7	1.15	0.04	2	0.59	0.02			
Cutler et al.	*A/J*	33	4.20	0.16	13	0.94	0.04			
"	*AKR/J*	22	3.39	0.13	18	1.41	0.05			
"	*BALB/cJ*	36	5.79	0.23	28	6.39	0.25			
"	*C3H/HeJ*	25	3.68	0.14	11	0.77	0.03			
"	*CBA/J*	29	4.15	0.16	18	2.04	0.08			
"	*DBA/2J*	24	3.92	0.15	7	0.60	0.02			
"	*LP/J*	26	4.56	0.18	9	1.45	0.06			
"	Mean	27	4.24	0.17	14	1.94	0.08			
Henrichsen et al.	*A/J*	179	14.30	0.56	28	3.21	0.12			
"	*AKR/J*	136	10.37	0.40	25	2.47	0.10			
"	*BALB/cJ*	-	-	-	-	-	-			
"	*C3H/HeJ*	-	-	-	-	-	-			
"	*CBA/J*	-	-	-	-	-	-			
"	*DBA/2J*	161	13.57	0.53	31	3.14	0.12			
"	*LP/J*	158	12.62	0.49	44	5.18	0.20			
"	Mean	158	12.72	0.49	32	3.50	0.14			
Cahan et al.	*A/J*	138	2.51	0.10	32	0.83	0.03	140	15.82	0.62
"	*AKR/J*	159	2.32	0.09	20	0.81	0.03	143	18.42	0.72
"	*BALB/cJ*	-	-	-	-	-	-	-	-	-
"	*C3H/HeJ*	135	2.02	0.08	31	0.79	0.03	139	15.93	0.62
"	*CBA/J*	-	-	-	-	-	-	-	-	-
"	*DBA/2J*	156	2.49	0.10	24	0.80	0.03	135	17.95	0.70
"	*LP/J*	-	-	-	-	-	-	-	-	-
"	Mean	147	2.33	0.09	26	0.81	0.03	139	17.03	0.66

Only CNVs found in strains studied here have been included. Experimental designs differed: Graubert et al. [23] used 20 inbred test strains, with one animal per strain, conducted their experiments on a NimbleGen 385K array, and used CBS [35] to detect CNVs; Cutler et al. [14] tested 41 inbred strains, used two animals per strain (in dye swap replicates), conducted their experiments on an Agilent 244K array, and used the detection algorithm GLAD [38]; Henrichsen et al. [5] analyzed 12 inbred strains (as well as 21 wild mice), tested three individuals per strain for CNVs, also employed a NimbleGen 385K array, and used an in-house HMM to detect CNVs; finally, Cahan et al. [6] tested 19 inbred strains, pooling the DNA of two to six animals per strain, conducted their experiments on a NimbleGen 2.1M array, and used wuHMM [15] for CNV detection. Note that Cahan et al. [6] classified CNVs as ‘gain’, ‘loss’ and ‘abnormal’, and that the copy number status of abnormal CNVs was not published.

### Biological Characteristics of CNV Regions

We analyzed the genomic content and functional impact of CNV regions. In line with a previous study [6], we classified CNV regions by length (small (<10 Kb), medium (10–100 Kb) and large (>100 Kb)), and then characterized their repetitive sequence content.

Since one proposed mechanism for CNV formation is non-allelic homologous recombination (NAHR) [21], sequence features that are recombination substrates may act as CNV nurseries. We assessed CNV region content for such features and tested for significant enrichment by permutation; results are given in Table S6. We found that segmental duplications (SDs) and long tandem repeats (LTRs) are enriched within and around medium and large CNV regions, and that long interspersed repetitive elements (LINEs) are enriched within them, and also in the flanking sequence of large CNV regions. Conversely, short interspersed repetitive elements (SINEs) are depleted in CNV regions >10 Kb. These results are largely in agreement with previous analyses [6], as were our results for short CNV regions: LINEs are depleted in them; SDs are depleted within and around them; whilst LTRs and SINEs are neither enriched nor depleted.

A Gene Ontology (GO) enrichment analysis produced very similar results to those obtained for human-CNV genes [22] and for mouse-CNV genes [14] (Table S7). Specifically, genes involved with immunity (for example: “antigen binding”, “defense response”, “immune response” and “antigen presentation by MHC class I”) are all enriched in CNV regions, as are those related to environmental sensory (“odorant binding” and “pheromone binding”). In contrast, genes related to basic cellular processes (“nucleus”, “DNA binding” and “protein binding”) are all significantly under-represented in CNV-region genes.

We also analyzed the effect of copy number variation on gene expression using genome-wide expression data in three tissues (brain (hippocampus), liver and lung), from the inbred strains [19]. In agreement with previous studies [5], we found that the expression variance of transcripts mapping within CNV regions is greater than that of transcripts mapping elsewhere on the genome (Table S8), and that CNVs affect transcript expression levels by altering transcript dosage, although this was not always the case (Table S9 and Figure S1).

### Comparison with Previously Reported CNVs

Tables 2 and 3 report respectively comparisons of CNV numbers and replication rates between this study and four other genome-wide experiments [5], [6], [14], [23]; Table S10 collates the data for all the CNVs from these five studies. Using our CNVs we attempted to quantify factors that give rise to variation between studies.

**Table 3:** Fraction of CNVs, from each study, replicated by CNVs in other studies.

Deletions					
	Graubert	Cutler	Henrichsen	Cahan	Agam
% Graubert et al.		60.3	88.7	67.7	79.3
% Cutler et al.	17.0		44.3	56.8	61.5
% Henrichsen et al.	5.6	15.0		32.3	33.9
% Cahan et al.	1.3	3.5	8.6		29.8
% Agam et al.	2.5	6.8	20.1	38.2	
Gains					
	Graubert	Cutler	Henrichsen	Cahan	Agam
% Graubert et al.		59.4	82.4	100	100
% Cutler et al.	7.9		38.6	66.4	41.3
% Henrichsen et al.	10.2	22.7		39.9	27.3
% Cahan et al.	2.1	15.1	6.9		25.2
% Agam et al.	3.6	8.4	12.9	23.3	
Abnormal CNVs					
	Graubert	Cutler	Henrichsen	Cahan	Agam
% Cahan et al.	4.1	9.6	22.9		46.3

Each row gives the percentage of CNVs from one study replicated by each of the remaining studies (columns); for example, 17.0% of deletions identified by Cutler et al. [14] were also identified by Graubert et al. [23], whereas 60.3% of deletions identified by Graubert et al. [23] were also found by Cutler et al. [14]. In each pairwise comparison, only the CNVs from strains common to both studies were included. A CNV was counted as replicated if it had at least partial overlap with a CNV on the same strain in another study. Note that, because Cahan et al. [6] has a classification for abnormal CNVs separate from deletions and gains, we used the sum total including abnormal CNVs to calculate the fraction of deletions and gains in any one study which are replicated by this study [6].

First, to establish an upper bound on the reproducibility of aCGH, we compared the CNV calls in our initial experiments to those obtained in technical replicates (for each strain we repeated each experiment in dye swap using DNA from the same animal). In the remainder of this section we refer to the overlap between two sets of CNVs as the number of CNVs in their intersection, divided by the number of CNVs in their union; hence there was a mean overlap of 0.44 for deletions (variance  = 0.0051, min  = 0.37, max  = 0.57), and 0.50 for gains (variance  = 0.0077, min  = 0.37, max  = 0.62) between technical replicates.

We examined the effects on reproducibility of changing the biological sample, CNV detection algorithm, and microarray platform. We assessed each factor, while the others were fixed. Assessing differences in biological samples is simplified in mouse analyses due to the availability of inbred strains. We compared CNVs called in animals of the same strain using the same platform and algorithm (data published by Henrichsen et al. [5]), and found a mean overlap of 0.43 for deletions (variance  = 0.013, min  = 0.15, max  = 0.59) and 0.25 for gains (variance  = 0.020, min  = 0.056, max  = 0.60). Thus technical replicates (i.e. dye-swap experiments) produce more consistent results than biological replicates.

To establish the effect of changing CNV detection algorithms, we compared the CNV calls published by Cahan et al. [6] to the putative CNVs that we detected in their raw data using SW-ARRAY; this gave a mean overlap of 0.49 for all CNVs (variance  = 0.0019, min  = 0.44, max  = 0.54). We were unable to calculate separate results for deletions and gains because not all of the CNVs published by Cahan et al. [6] were categorized as such.

Finally, we considered the effect of changing platforms by comparing our CNVs to those obtained in an earlier experiment in which we used a lower density NimbleGen array with ∼385,000 probes (385K array), but with DNA from the same animals and employing SW-ARRAY for CNV detection (Methods S1, Table S11). In that experiment we found 121 deletions and 48 gains, with median lengths 178.3 Kb and 126.7 Kb respectively. CNVs from the lower density array had a high concordance with the 2.1M array CNVs (85.1% of deletions and 81.6% of gains were replicated), but since the high-density array detected so many more CNVs the overlap between the experiments is low (0.07 for deletions (variance  = 0.0006) and 0.08 for gains (variance  = 0.0004)). Almost all the non-replicated CNVs (98.7% of singleton deletions and 98.0% of singleton gains) were found by the 2.1M array.

### Molecular Validation

We next assessed the false positive rates in aCGH by independent molecular validation of a subset of CNV regions (44 deletions and 17 gains). To do so, we used a combination of PCR, sequencing, real-time PCR, FISH and MLPA (Table S12). We found that 14 of 44 deletions (32%), and 6 of 17 gains (35%), were false positives.

**Figure 3:**
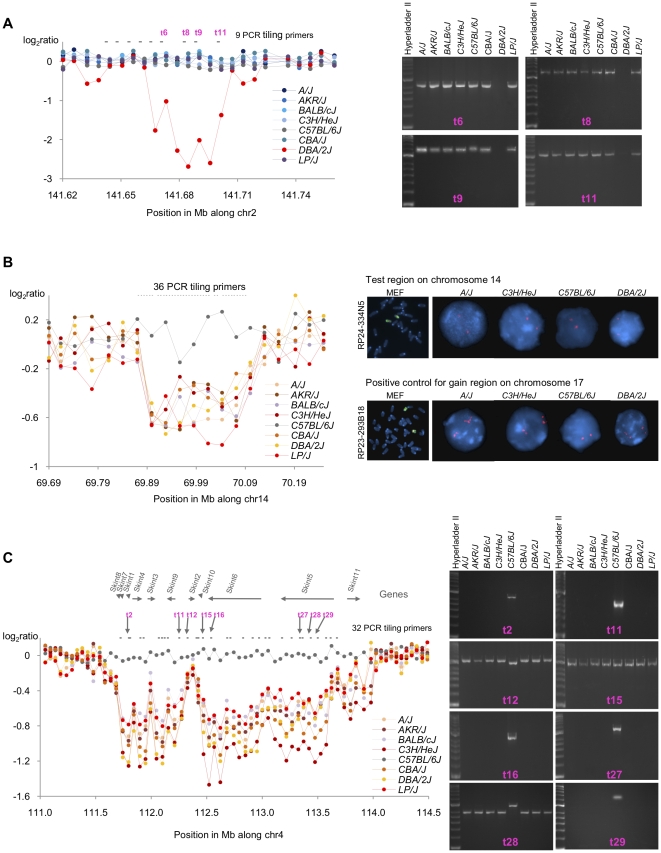
Example of a simple, false positive and complex CNV region. Here we show an example for each class of deletion-CNV region: A) simple, B) false positive and C) complex. Graphics on the left hand side show the distribution of CGH signal intensities in eight inbred strains of mice: *A/J*, *AKR/J*, *BALB/cJ*, *C3H/HeJ*, *C57BL/6J*, *CBA/J*, *DBA/2J* and *LP/J*. The X-axis is the position in Mb and the Y-axis is the log_2_ ratio (averaged in windows of 5 probes for A and B and 40 for C). Images on the right hand side constitute a representative set of independent validation experiments. **A**: 39 Kb simple deletion identified in *DBA/2J* on Chr 2: 141.669 Mb–141.708 Mb. Tiling PCR primers are displayed at the top of the graph. In total, we designed 9 tiling primers (t1 to t11), each amplifying regions of ∼1.2 Kb across the region and its 5′ flanking region. We highlight a representative set in pink (primers t6, t8, t9 and t11) for which PCR results are shown on the right of the graphic. See Table S12 for details of all primers and PCR results. This deletion lies within intron 8 of the gene Macrod2 [36]. **B**: 220 Kb false positive deletion identified in all the test strains, on Chr14: 69.87 Mb–70.09 Mb, in our 385K aCGH study. We designed 36 tiling primers spanning the region (represented at the top of the graphic). PCR results showed amplification in all 8 strains (Table S12) suggesting a false positive deletion. We also carried out FISH experiments. We used two BACs, RP24-334N5 (Chr 14: 69.9 Mb–70.07 Mb) for the test region on chromosome 14 and RP23-293B18 (Chr 17: 30.83 Mb–31.0 Mb) as a positive gain control on mouse chromosome 17. FISH data show that the region is not deleted. **C**: 2.44 Mb fragmented deletion identified in all the test strains on Chr 4: 111.58 Mb–114.02 Mb. PCR results are shown for 8 representative tiling primers out of a total of 32 (highlighted in pink). There is no amplification in the test strains from t2 to t11, t16 to t27 and t29, thus validating the deletion. However, primers t1, t12 to t15 and t28 have amplified in all strains. Genes are represented at the top using grey arrows. The first deletion affects Skint4, Skint3 and Skint9 [37], and the second deletion affects Skint6 and Skint5; the latter finding has not been reported previously.

Molecular characterization revealed an unexpected feature of the validated CNV regions. In 21 of 61 cases (34%; 16 deletions and 5 gains), PCR results from sequential sites within the CNV region indicated that the whole segment was either deleted or gained. We refer to CNV regions with this pattern of results as simple. However 19 of 61 CNV regions (31%; 13 deletions and 6 gains) consist of smaller CNVs interspersed with non-CNV segments, or contain mixed segments of gains and deletions; we classify such CNV regions as complex (Figure 3).

**Figure 4:**
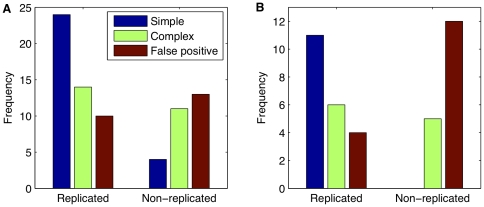
Concordance between studies for simple, complex and false positive CNVs. **A**: Deletion-CNV regions detected in our experiments were determined, by molecular validation, to be either simple (blue), complex (green) or false positive (red); their constituent CNVs were classified accordingly. Examining only the CNVs detected in the four strains which were common to both our study and the Cahan et al. study [6] (*A/J*, *AKR/J*, *C3H/HeJ*, *DBA/2J*), we established, for each category, the frequency of our deletions replicated and not replicated; a CNV was counted as replicated if it had at least partial overlap with a CNV on the same strain in that study. **B**: Similarly for gains.

The distinction between simple and complex CNV regions correlates with reproducibility between studies; simple CNVs were easier to detect on aCGH than complex CNVs. The majority of our simple CNVs were found in another study [6], but this was not the case for complex CNVs or false positives (χ^2^ test of 2×3 contingency table yielded *P*  = 0.005 (χ^2^ = 10.5, d.f.  = 2) for deletions, and *P* = 0.001 (χ^2^ = 14.8) for gains) (Figure 4).

**Figure 5:**
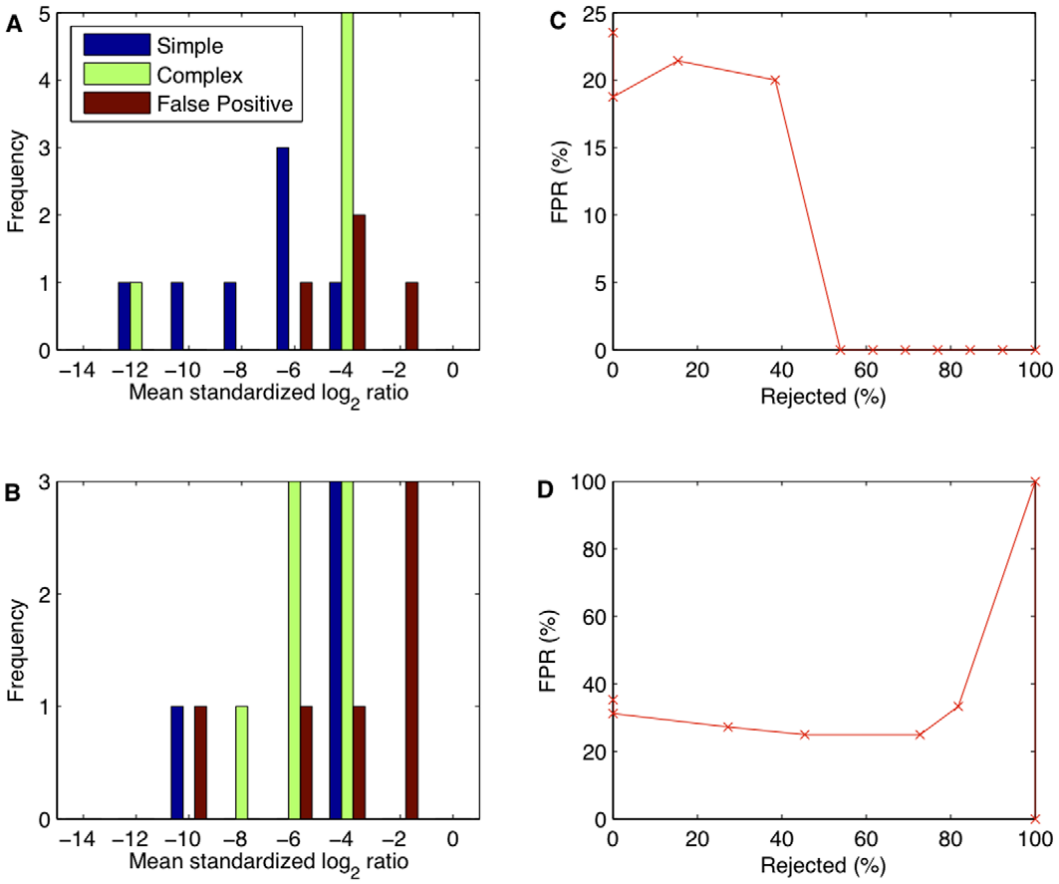
Distribution of mean standardized log_2_ ratios in simple, complex and false positive deletions. **A**: Distribution of the mean standardized log_2_ ratios in simple (blue), complex (green) and false positive (red) *A/J* deletions that were included in the molecular validation experiments described in the main text. **B**: Similarly for *BALB/cJ*. **C**: We examined all thresholds for accepting a deletion between −1 and −14. For each threshold we plot the false positive rate (FPR), calculated as the percentage of all accepted deletions that are false positives, against the percentage of all verified deletions that are rejected (where verified deletions are defined as those which were categorized as either simple or complex in the molecular validation experiments). Results are shown for *A/J*. **D**: Results are shown for *BALB/cJ*. (See Figure S2 for the remaining test strains).

We investigated whether the rates of false positive detection could be improved by applying a stringent threshold to the log_2_ ratios, as reported in Cahan et al. [6]. To do this, we focused on the set of deletion-CNV regions in our molecular validation pipeline and examined each deletion's mean standardized log_2_ ratios. These deletions were grouped by strain, and then categorized as ‘simple’, ‘complex’ and ‘false positive’, depending on the molecular validation result of the corresponding CNV region (Figure 5A and B, and Figure S2 A to E). We found that false positive deletions can have large negative mean standardized log_2_ ratios, suggesting that this is not a good indicator of the accuracy of a CNV call.

In five of the seven test strains (*A/J*, *AKR/J*, *CBA/J*, *DBA/2J* and *LP/J*) it was possible to find a threshold for the standardized log_2_ ratios that yielded a 0% false positive rate, but this entailed rejecting more than 50% of the verified deletions (that is, those deletions determined to be either simple or complex by molecular validation: Figure 5C and Figure S2 F to I); in the remaining two test strains (*BALB/cJ* and *C3H/HeJ*) the false positive deletions have the largest negative mean standardized log_2_ ratios, so the threshold required for a 0% false positive rate also rejects all verified deletions (Figure 5D and Figure S2 J).

**Figure 6:**
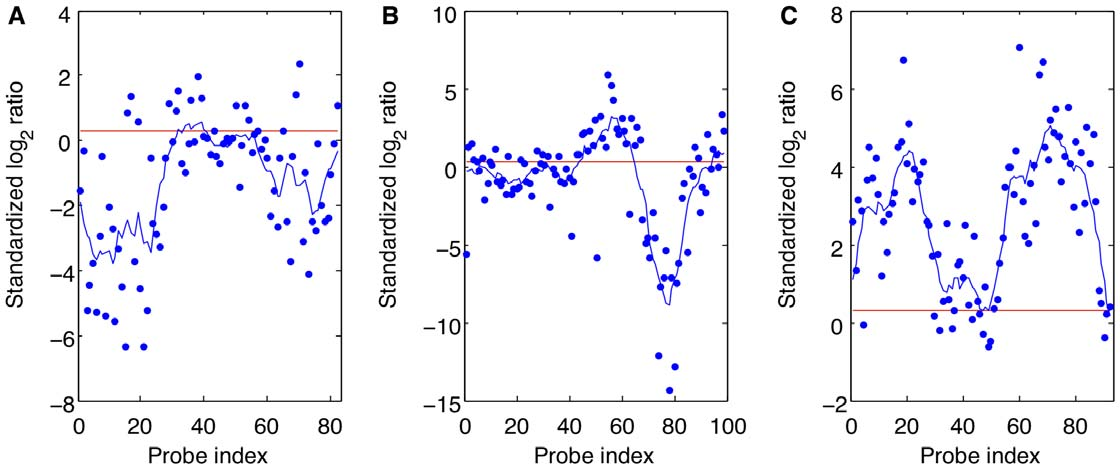
Complex CNV architecture elucidated by targeted high-density aCGH. Three CNVs in *C3H/HeJ*, inspected using the targeted array; the red line is the median log_2_ ratio observed in control regions, blue dots are the SNP standardized log_2_ ratios for each probe in the region, and the blue line is the smoothed signal (using a window size of 10% of the number of probes). **A**: Putative deletion on Chr 1: 95.74 Mb–95.89 Mb is composed of two smaller deletions, separated by a region of normal copy number. **B**: Putative deletion on Chr 16: 49.34 Mb–49.37 Mb harbors a small gain. **C**: Putative gain on Chr 18: 31.78 Mb–31.80 Mb is composed of two smaller gains separated by a region of normal copy number.

Finally, we used a very high-density array (mean probe spacing of 214 bp) to interrogate 241 deletion-CNV and 105 gain-CNV regions identified by the 2.1M array across the 7 test strains. We excluded 15 regions where there is an overlap between deletions and gains on different strains. The targeted array classified approximately 30% of these CNVs as complex (Figure 6). We then used the data to estimate the false negative rate in our experiment. For each strain we determined the structural variant present (or absent) in each of the targeted CNV regions, irrespective of whether the strain in question carried a corresponding CNV according to the 2.1M array experiment. Since we expected the very high density array to be more accurate than the 2.1M array, we assumed that the CNVs detected there were a more accurate assessment of whether a CNV was present; hence we estimated the false negative rate by counting the number of regions in which the targeted array detected structural variation, but where the 2.1M array did not. On average, each strain had 190 targeted regions containing structural variation, 54.5% of which had not given rise to a CNV signal in the 2.1M array experiment.

We attempted to confirm this false negative rate using our molecular validation data. Each time we used PCR to validate a CNV call from the 2.1M array we checked whether that CNV was present or absent in all seven strains. The array might for example have identified a CNV in strain *AKR/J* but not in *A/J*, and we can therefore check not only whether the CNV is indeed present in *AKR/J* (false positive) but also whether it is missing in *A/J* (false negative). Using this approach, we found a mean false negative rate of 14.5% per strain for deletions and 19% per strain for gains.

## Discussion

Using a large data set of CNVs discovered in strains of inbred mice, we have quantified factors that contribute to discrepancies between aCGH studies. First, by comparing technical replicates from one experiment we have shown that the baseline reproducibility of aCGH experiments is low, consistent with a previous comparison of different array platforms [24]. Second, the choice of CNV detection algorithm makes a smaller but still significant contribution to discordance between studies than the combined effects of low reproducibility of aCGH and different biological samples. Third, platforms with very similar protocols for probe design and hybridization can produce highly concordant results (more than 80% of CNVs detected in the 385K array data were recapitulated in the 2.1M data), but probe density is a limiting factor for the detection of small CNVs, making it very hard to draw conclusions from comparisons between CNV sets detected using platforms of widely differing resolutions. Finally, molecular validation of CNVs, using a variety of independent methods, indicated that approximately one third of the CNVs detected by the 2.1M array were false positives.

Of the four mouse aCGH experiments reviewed here only one [6] used independent experimental techniques (PCR and real-time PCR) to validate more than ten of their putative CNVs (61 of 3,359). Across all the experiments we reviewed, the average number of PCR primers used to validate each CNV was less than 2 (Table S13). We validated 61 CNV regions, with a mean of 7 PCR primers per region. We note that other studies have used very high-density arrays to validate CNV regions [5], but consider that this technique is less informative than PCR-based assays because it is prone to the same confounds as the original experiments, discussed above.

With this caveat on the use of high-density arrays for determining the accuracy of CNV calls from array based experiments, we tackled the more difficult problem of identifying false negatives using a targeted array to examine regions where no CNV had been called by the 2.1 M array. The array comparison identified a false negative rate of 54.5%, a figure that we attempted to corroborate using data from our molecular validation experiments. The latter indicated a false negative rate of 14.5% for deletions and 19% for gains, substantially lower than those obtained from the between-array comparison. However we believe the array-comparisons figure is a better estimate, because the molecular validation strategy only interrogates a small fraction of the region predicted to have a CNV. Since many CNVs are complex, with regions of deletion or gains interspersed with regions of non-CNV DNA, a proportion of the PCR validations will, by chance, have missed the CNV. This will have resulted in an underestimate of the false negative rate. Thus the molecular validation result can be regarded as a lower bound on the false negative rate. Additional support for the 54.5% figure comes from the biological features of our CNVs, which are also found by others. The commonality suggests that many of the CNV calls found in different studies are correct, true positives, and that the low concordance between publications must therefore be attributed to large numbers of false negatives.

One source of variation between studies is likely to be the presence of CNVs segregating within inbred strains. For instance a survey of only five chromosomes at relatively low resolution identified CNVs segregating in *C57BL/6J* mice [25]. Since most studies use only a few animals from each strain and do not look at pedigrees, the full impact of this source of variation has yet to be quantified.

Our results help explain why aCGH experiments differ, and also how those differences can be avoided. First, we show that the effects of known SNPs in probes can be ameliorated by a novel but simple pre-processing step that accounts for SNP content, and allows us to retain affected probes in the analysis. We expect the results will be improved once a complete SNP catalogue is available. Second, technical and biological variation is a major cause of discrepant findings. Assuming the sources of this variation are random, simply repeating the experiment enough times should reduce the error. Consequently CNVs found by multiple studies are more likely to be true positives. However our work suggests that there is another source of variation, whose importance has not hitherto been fully appreciated, that complicates this simple solution.

Low concordance between studies is in part due to the molecular nature of CNVs. We have found that many CNVs consist of a series of smaller deletions and gains interspersed by regions where the DNA copy number is normal [26], [27]. Discrepancies between studies are more likely to occur when detecting these complex CNVs, compared to the simpler deletions and gains. This is because hybridization signals from multiple probes are used to detect a CNV and so, within a complex CNV, only a fraction of probes are likely to detect changes in genome content. Arrays interrogating the same CNV region in the same individual would be expected to yield inconsistent results if the probes are in different locations; in one case they identify copy number changes while in the other they may not. ‘False positives’ may therefore be true positives that array probes were incorrectly placed to detect.

Our dichotomous classification of CNVs into simple and complex, though useful in interpreting aCGH data, may be artefactual. Simple CNVs do not appear to possess a unique biological identifier. We could find no sequence feature, strain distribution patterns of sequence variants, or hybridization signature that would enable their unambiguous identification. Furthermore, data from dense arrays reveal a spectrum of CNVs [1], from small insertion-deletions to megabase scale structural variants. Complex CNVs may simply be the coincidental co-localization of smaller, simpler CNVs, whose presence is more likely to perturb the aCGH hybridization profile than an isolated, small copy number alteration. The detection and characterization of complete sets of CNVs will require the application of next generation sequencing which will doubtless reveal yet more unexpected features of the molecular nature of structural variation across the genome.

## Materials and Methods

### Mouse DNA Samples

DNA of male mice from eight inbred strains (*A/J*, *AKR/J*, *BALB/cJ*, *C3H/HeJ*, *C57BL/6J*, *CBA/J*, *DBA/2J* and *LP/J*) was purchased from the Jackson Laboratory (JAX, http://www.jax.org) at a concentration of 1 µg/µl. DNA was diluted 1 in 5, giving a working concentration of 200 ng/µl. 50 µl was sent to NimbleGen (Iceland) for aCGH work.

### NimbleGen 2.1 Million Probe Array

We conducted a comparative genomic hybridization experiment using a NimbleGen long-oligonucleotide array containing ∼2.1 million probes. The probes are 50–75mers selected from a *C57BL/6J* Build 37 tiling database. They span all chromosomes, are evenly spaced, and have been designed to be isothermal (as far as possible) to ensure uniform hybridization behaviour. Considering only the autosomal chromosomes, there were 1,967,439 probes with mean, median, 90^th^ and 99^th^ percentile spacing of 1,228, 1,135, 1,206 and 3,574 bp, respectively. The maximum spacing between probes is 7 Mb on chromosome 7: 39–46 Mb, where there is a gap in the *C57BL/6J* sequence.

### Array Processing and CNV Detection

Normalization of hybridization signals was performed by NimbleGen using standard protocols. We removed probes whose sequences contained repeats as identified by RepeatMasker [28], leaving 1,748,617 probes with a mean spacing of 1,381 bp, and 50^th^, 90^th^ and 99^th^ percentile spacing of 1,136, 1,832 and 6,679 bp (Materials S1, Figure S3).

Within each strain, probes were annotated for SNP content (using only the sequence variants between the strain and *C57BL/6J*) based on the Perlegen Sciences SNP set [18]. Then log_2_ ratios were grouped together according to the number of SNPs in their corresponding probe (zero, one, two and more than two). To measure the effect of SNPs on probe hybridization a linear regression analysis was conducted with SNP content and log_2_ ratio as the explanatory and dependent variables, respectively. Finally, the distribution of log_2_ ratios in each group was standardized by subtracting the group's median from each log_2_ ratio, and then dividing by the group's median absolute deviation.

CNVs were called using SW-ARRAY [20] with deletion and gain thresholds set at the 10^th^ and 90^th^ percentiles of the standardized distribution of log_2_ ratios within each strain. Only CNVs significant at a genome-wide 5% significance threshold (determined by permutation) were reported. Then we applied a post-processing step to remove CNVs with a low probe density (Materials S1). Finally, each comparison between a test strain and *C57BL/6J* comprised a normal and dye swap hybridization using DNA samples from the same animal; we processed each independently using the above pipeline, and then only those CNVs that were at least partially replicated in both experiments were reported.

### Permutation Testing for Significance of CNV Region Genomic Content

We have observed that CNV regions cluster (Materials S1, Figure S4). Therefore simply permuting CNVs randomly across the genome (as has been done previously [6]) is inappropriate because the clustering is lost. We used a novel method for CNV region permutation that maintains clusters called rotational permutation (Materials S1, Figure S5).

Using this method we generated 1000 permuted CNV region sets. Then, for each biological feature of interest, we generated an empirical null distribution of its overlap with CNV regions by calculating and recording the percentage of CNV region bases that overlapped it in each of the permuted sets. We then calculated the percentage of real CNV region bases that overlapped the feature, and compared this to the null distribution to obtain a *P*-value.

### Gene Ontology Enrichment Analysis

We used the pipeline for GO enrichment analysis of CNVs in Nguyen et al. [22]; the likelihood that a GO annotation is over- or under- represented among CNVs is estimated using the hypergeometric distribution, and then the false discovery rate (FDR) is controlled (here it was kept at 5%) to select the most significant results.

### Calculating Overlap between CNV Sets

Determining the overlap between two sets of CNVs was a two step procedure:

- CNVs located on the same strain in both experiments, and which had at least partially overlapping genomic coordinates, were grouped into CNV regions; CNVs that were only detected in one of the experiments became singleton CNV regions.
- The overlap statistic was calculated as the number of CNV regions containing more than one CNV, divided by the total number of CNV regions.

### Analysis of Cahan et al. Array CGH Data

Array CGH data from the study published by Cahan et al. [6] were downloaded from the NCBI GEO website (http://www.ncbi.nlm.nih.gov) using accession code GSE10656. For each of our test strains included in that study (*A/J*, *AKR/J*, *C3H/HeJ* and *DBA/2J*), we extracted the normalized log_2_ ratios (rather than using the raw intensity data directly). We performed our SNP standardization before applying SW-ARRAY to detect CNVs; note that probes with a high repeat content were not removed for this analysis. We chose stricter thresholds for SW-ARRAY (the 5^th^ and 95^th^ percentiles of genome-wide log_2_ ratios for deletions and gains, respectively) than we had for own data; doing so reduced the number of CNVs compared to those obtained with thresholds set at the 10^th^ and 90^th^ percentiles, and improved their concordance with the published CNVs [6]. We kept all CNVs significant at a genome-wide 5% significance threshold, regardless of their probe density.

### PCR, Sequencing and Multiplex Ligation Probe Amplification (MLPA)

Primers were designed using Primer3 [29] and purchased from MWG (Germany). Three independent PCR reactions were carried out with Hotstar Taq obtained from Qiagen (Germany). Reactions were performed as previously described [30]. PCR products were purified in a 96-well Millipore purification plate resuspended in 30 µl of H_2_O and sequenced as previously described [30]. All sequencing reactions were run out on an ABI3700 sequencer and assembled by using PHRED/PHRAP [31]. Consed was then used for editing and visualization of the assembly [32]. For quantification of gene relative copy number, we used the comparative Ct method [33]. The Ct values for each set of triplicates were averaged. Ct values were normalized against a control primer. The number of copies for each strain was calculated as 2∧(normalized Ct for test strain – normalized Ct for reference strain). MLPA primers were designed to hybridize to regions without sequence polymorphisms and MLPA was performed using published protocols [34]. Internal controls from regions with a CNV were included in the MLPA analyses. Data were analyzed using Applied Biosystems Peak Scanner software and MRC-Holland Coffalyser software (http://old.mlpa.com/coffalyser).

### Fluorescence In Situ Hybridization (FISH)

Bacterial artificial chromosome (BAC) clones mapping to the relevant regions of the genome were purchased from Geneservice (Cambridge, UK; http://geneservice.co.uk). All BACs are derived from inbred strain *C57BL/6J*. BACs were prepared and hybridized to mouse chromosomes as previously described [30].

### Targeted High-Density Array

NimbleGen designed a targeted array to interrogate 348 CNV regions (241 deletions and 107 gains) at a probe density of approximately one every 214 bp. In addition, ten 50 Kb negative controls (regions where there are no known CNVs in any of the test strains) were included on the array. Only probes with no repetitive sequence content were allowed. We standardized the data to account for probe SNP content, as described for the 2.1M array above. Then, for each test strain, we analyzed each CNV region as follows:

- We segmented the data from the region using the MATLAB Bioinformatics Toolbox implementation of Circular Binary Segmentation ([35], http://www.mathworks.com/access/helpdesk/help/toolbox/bioinfo/ref/cghcbs.html), with the default parameters.
- Then we tested each segment using a method similar to that proposed by Henrichsen et al. [5]: we used the Mann-Whitney U test to determine whether the standardized log_2_ ratios from probes in the segment were significantly different from those obtained in the control regions (*P*<0.05); if they were, we declared the segment to be a CNV if the median standardized log_2_ ratio in the segment was >2 x s.d.(median standardized log2 ratios in control regions).
- If we determined that at least one segment of the region was a CNV, then we declared the whole region to be copy number variant in the strain of interest.

## Supporting Information

Figure S1 Correlation between transcript CNV status and expression. For each strain, in each tissue, box and whisker plots of the normalized relative expression (calculated as the ANOVA logP, see Table S8 legend) for all differentially expressed transcripts are shown. Transcripts are classified according to their CNV status: deletion (black), non-CNV (grey), or gain (white). The number of probe sets in each sample is shown under the boxplots.
(0.49 MB DOC)

Figure S2 Distribution of mean standardized log_2_ ratios in simple, complex and false positive deletions. A-E: Distribution of the mean standardized log_2_ ratios in simple (blue), complex (green) and false positive (red) deletions in *AKR/J, CBA/J, DBA/2J, LP/J* and *C3H/HeJ*, respectively. F-J: We examined all thresholds for accepting a deletion between -1 and -14. For each threshold we plot the false positive rate (FPR) against the percentage of all verified deletions that are rejected. Results are shown in the same strain order as for plots A-E.
(0.64 MB DOC)

Figure S3 Relationship between probe repeat content and hybridization. A: Box and whisker plots of the log_2_ ratios from probes in the 2.1M array *A/J* versus *C57BL/6J* normal dye experiment. Probes are grouped by repeat content. Probes either have no repetitive sequence at all, or they are found to have a minimum of 33% (this is due to the algorithm and default settings used by RepeatMasker). B: Chromosome 1 log_2_ ratio profile, with repetitive probes highlighted in green. Such probes constitute 11.1% of all probes.
(0.06 MB DOC)

Figure S4 Distributions of observed and expected inter-CNV region distances. A: Histograms of the two distributions, with observed values in blue and expected in red. B: QQ-plot of the distributions.
(0.30 MB DOC)

Figure S5 Schematic diagram of rotational permutation. 1: Start with CNV regions on a genome. The start and end of the genome are delimited by vertical green lines, the chromosomes by blue lines, and the CNV regions by red rectangles. 2: Wrap the genome into a circle. 3: Rotate the CNV regions by a random number of bases. 4: Unwrap the genome so that it is possible to measure the overlap with the biological attribute of interest.
(0.15 MB DOC)

Table S1 Perlegen SNPs. Number of autosomal SNPs identified by Frazer, et al. in the seven test strains, when compared to *C57BL/6J*.
(0.02 MB XLS)

Table S2 2.1M array probes with SNP content. For each strain, the total number of probes which contain SNPs is listed. Subtotals are given for probes with one, two, three or four annotated SNPs. Results are shown as a percentage of the total number of 2.1M probes considered in this analysis.
(0.03 MB XLS)

Table S3 SNP effect depends on position within probe. Log_2_ ratios of probes containing one SNP were classified by the position of the SNP within the probe. The log_2_ ratios from probes with central SNPs were compared to the log_2_ ratios from probes with edge SNPs using a Mann-Whitney U test. P-values from these comparisons are shown for each strain and experiment (d.s.  =  dye swap).
(0.02 MB XLS)

Table S4 CNV coordinates. Genomic coordinates are given for the putative CNVs detected in each strain. Median P-values from the SW-ARRAY analysis of the normal dye and dye swap experiments are given. Also shown are the mean and median standardized log_2_ ratios for each CNV, in both the normal dye and dye swap experiments.
(0.41 MB XLS)

Table S5 CNV region coordinates. CNV region coordinates and strain distribution patterns. Strains that harbour a CNV in a CNV region are denoted with a ‘1’.
(0.10 MB XLS)

Table S6 Enrichment and depletion of recombination substrates in CNV regions. Recombination substrates were downloaded from the University of Santa Cruz Genome Browser (http://genome.ucsc.edu) and re-mapped to Build 37 of the mouse genome, where necessary, using LiftOver (http://hgdownload.cse.ucsc.edu/downloads.html). (SD  =  segmental duplication, LINE  =  long interspersed repetitive element, SINE  =  short interspersed repetitive element and LTR  =  long tandem repeat.) CNV regions were categorized as short (<10Kb), medium (10 - 100 Kb), and long (>100 Kb), and were analyzed separately. CNV regions were permuted as described in the main text and Materials S1. Fold change was calculated as the percentage of CNV region basepairs overlapping the recombination substrate, divided by the expected percentage overlap (for each set of permuted CNV regions the percentage of CNV region bases in the substrate were recorded, and the expected percentage overlap was estimated as the median percentage over 1000 permutations.) P-values were calculated as described in the main text. Values shown in black text are for within and around the CNV region (up to 10 Kb away from the breakpoint), values in green refer only to enrichment (depletion) within regions, and values in red refer only in the neighboring segments. A ‘-’ indicates that no significant enrichment or depletion was detected.
(0.02 MB XLS)

Table S7 Statistically significant over- or under- representation of Gene Ontology terms in mouse-CNV regions. FDR is 5%. Note that any gene which is at least partially overlapped by a CNV region was included in this analysis.
(0.02 MB XLS)

Table S8 Expression variance of transcripts in CNV regions, in genomic segments near to CNV regions, and further away from CNV regions. We analysed the effect of CNV on gene epxression. We used genome-wide expression data in three tissues (brain (hippocampus), liver and lung), from 42 animals (five individuals from *A/J, AKR/J, BALB/cJ, C3H/HeJ, DBA/2J* and *C57BL/6J*, and four from *AKR/J, CBA/J* and *LP/J*), measured on Illumina expression arrays (Huang, et al. 2009). For each tissue we had a set of measured transcripts, and for each transcript the data consisted of its average expression level in each strain, and an ANOVA logP (that is, the negative, log_10_, P-value) measuring its differential expression across the eight strains. Median logPs are given for transcripts within CNV regions, within 250 to 450 Kb of CNV regions, 450 to 650 Kb away, and more than 650 Kb from the nearest CNV region breakpoint. P-values fron Mann-Whitney U tests that compare the logP values from one set of transcripts to those from all transcripts that are further away are also given.
(0.02 MB XLS)

Table S9 Comparing expression levels of transcripts in deletions, non-CNV regions, and gains. P-values from Mann-Whitney U tests, when expression levels of deletion transcripts are compared to those from non-CNV transcripts (P-value1), and when non-CNV transcripts are compared to gain transcripts (P-value2). Results are shown for each strain/tissue pair.
(0.02 MB XLS)

Table S10 Collated mouse CNVs from published aCGH studies. Genomic coordinates are given for the CNVs published in four aCGH based studies, as well as those published here. Where necessary, coordinates have been re-mapped to Build 37 using liftOver (http://genome.ucsc.edu/cgi-bin/hgLiftOver). Deletions, gains and complex CNVs (‘complex’ is defined in Cahan, et al. 2009) are given in three separate groups. Within each group CNVs are listed in alphabetical strain order, and within each strain they are listed in genomic order. For each CNV we show the originating study in which it was located, at the exact coordinates listed, and in the last six columns of the table we indicate which of the remaining studies detected any overlapping CNV, with matching directionality (gain or deletion), on that strain (minimum overlap  = 1 bp): ‘-1’ indicates that a strain was not included in a study; ‘0’ indicates no overlapping CNV; and ‘1’ indicates overlap. Note that because the type of complex CNVs (i.e., whether deletion or gain) detected in the Cahan, et al. 2009 study were not published, it was not possible to determine whether the overlap with these CNVs was also matched for direction. Therefore we annotate complex CNVs with the label ‘Cahan.complex’ in the ‘Originating Study’ column, and such CNVs are listed in the deletion and gain groups dependent on the types of CNVs which overlap them (so they will appear in both lists if they overlap deletions and gains in other studies). In addition there are two columns for the Cahan study: ‘Cahan’ and ‘Cahan.complex’; this is to distinguish overlap of a given CNV by a complex CNV (where matching direction could not be established) from overlap by a Cahan deletion or gain (where matched directionality to the CNV in question could be determined). Finally, the group of complex CNVs listed in the last part of the table are those which do not overlap with a CNV from any other study.
(1.53 MB XLS)

Table S11 Summary of CNVs found using the 385K array. Numbers and total sizes of and fraction of the genome covered by putative deletions and gains detected in each strain using the 385K array.
(0.02 MB XLS)

Table S12 Classification of CNV regions as simple/complex/false positive, primer details and PCR results. Column 1 gives the chromosome. Column 2 and 3 are the start and stop coordinates respectively, mapped onto Mouse Build37. Column 4 is the type of CNV region, either deletion or gain. Column 5 is the predicted SDP (*C57BL/6J* is always 0). Column 6 is the detection array (2.1M stands for the NimbleGen 2.1 million probe array, 385K for the NimbleGen 385,000 probe array). 2.1Ms refers to manual calls detected using NimleGen detection software SignalMap. Columns 7, 8, 9, 10 and 11 give numbers of PCR primers, real-time primers, sequenced fragments, MLPA primers and BACs used for FISH, respectively. Column 12 is the classification as simple, complex or false positive (as defined in the main text). NA is used when PCR data is not sufficient to classify the CNV region. Column 13 gives the primer name. A letter code is added at the end of the primer name: “rt” for real-time primers, “s” for fragments PCR'ed then sequenced and “mlpa” for MLPA primers. Column 14 is the primer sequence. Column 15 is the expected length of the amplicon (in bp). Column 16 gives the start coordinate of the primer in bp (Mouse Build37). Seven primers lying in a gain region on chromosome 8 (from 19,675,977bp to 20,032,624bp) failed to map onto mouse Build37 (the initial primer design was in Build36). And Column 17 (final column) is the average PCR result of the three independent reactions. 0 refers to no amplification in case of qualitative PCR and MLPA; and to an increase of copy number in case of quantitative PCR. The reference strain is always 1, referring to amplification and normal copy. For qualitative PCR, 2 is used to indicate amplification but with a different size amplicon than expected (for example when there is a short indel in the fragment). For quantitative PCR, 2 indicates a decrease in copy number. 13 primer pairs (out of 429) failed to amplify in three independent attempts.
(0.17 MB XLS)

Table S13 Experimental validation carried out by previous mouse CNV studies. Column 1 gives the reference of the mouse CNV study, column 2 is the array type used for the detection of the CNVs, column 3 gives the number of assessed CNV regions and column 4 gives the total number of primers used for independent validation.
(0.02 MB XLS)

Materials S1 (0.06 MB DOC)

Methods S1 (0.03 MB DOC)
