# Supplementary material for: Elusive Copy Number Variation in the Mouse Genome
Source: PLoS One. 2010 Sep 21;5(9):e12839. doi: 10.1371/journal.pone.0012839 (PMC2943477; doi:10.1371/journal.pone.0012839)
Supplement: Figure S1 — Correlation between transcript CNV status and expression. For each strain, in each tissue, box and whisker plots of the normalized relative expression (calculated as the ANOVA logP, see Table S8 legend) for all differentially expressed transcripts are shown. Transcripts are classified according to their CNV status: deletion (black), non-CNV (grey), or gain (white). The number of probe sets in each sample is shown under the boxplots. (0.49 MB DOC) [file pone.0012839.s001.doc]

**Figure S1 – Correlation between transcript CNV status and expression.**


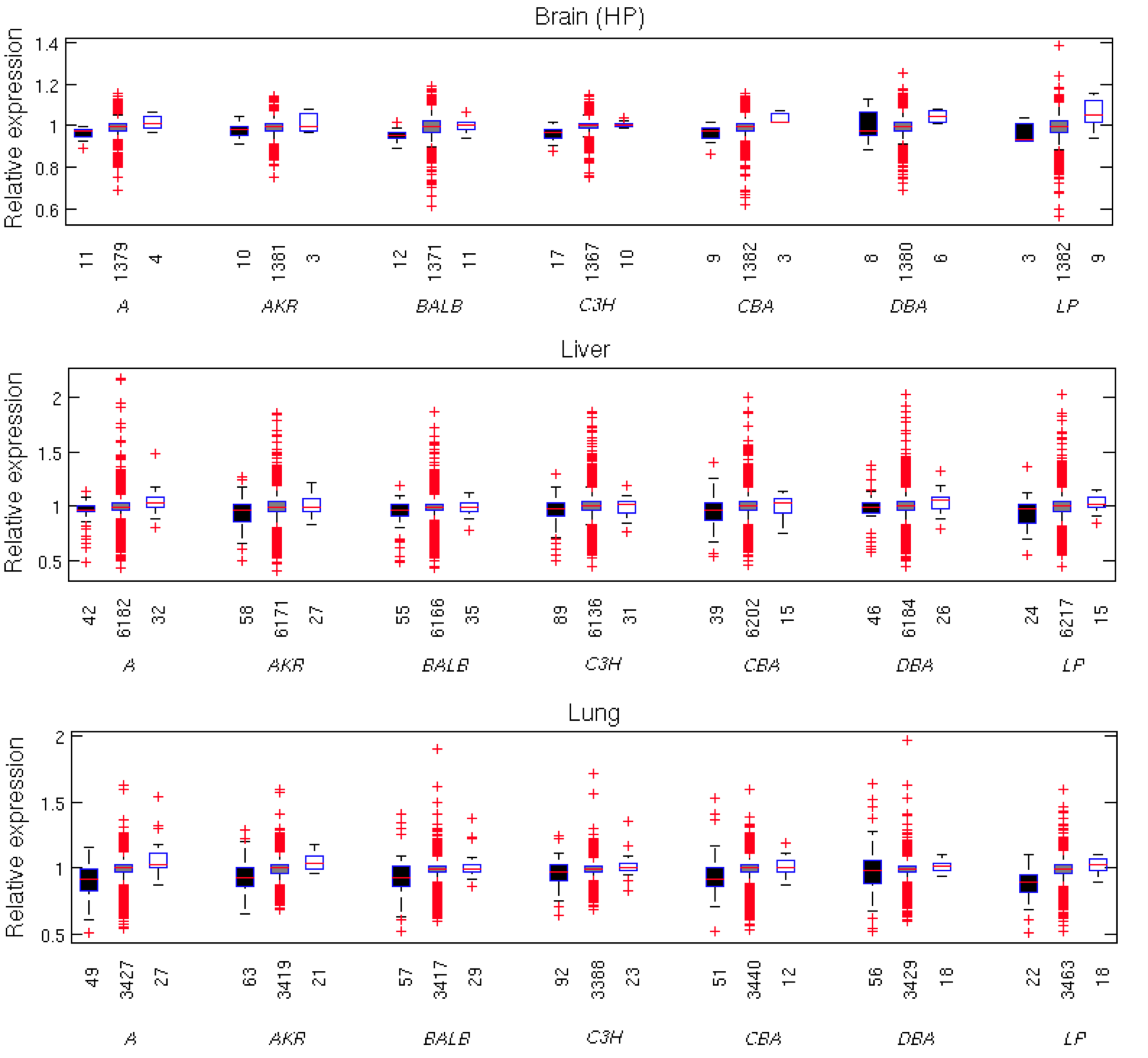


For each strain, in each tissue, box and whisker plots of the normalized relative expression (calculated as the ANOVA logP, see Table S8 legend) for all differentially expressed transcripts are shown. Transcripts are classified according to their CNV status: deletion (black), non-CNV (grey), or gain (white). The number of probe sets in each sample is shown under the boxplots.
