# Supplementary material for: Elusive Copy Number Variation in the Mouse Genome
Source: PLoS One. 2010 Sep 21;5(9):e12839. doi: 10.1371/journal.pone.0012839 (PMC2943477; doi:10.1371/journal.pone.0012839)
Supplement: Figure S2 — Distribution of mean standardized log2 ratios in simple, complex and false positive deletions. A-E: Distribution of the mean standardized log2 ratios in simple (blue), complex (green) and false positive (red) deletions in AKR/J, CBA/J, DBA/2J, LP/J and C3H/HeJ, respectively. F-J: We examined all thresholds for accepting a deletion between -1 and -14. For each threshold we plot the false positive rate (FPR) against the percentage of all verified deletions that are rejected. Results are shown in the same strain order as for plots A-E. (0.64 MB DOC) [file pone.0012839.s002.doc]

**Figure S2 – Distribution of mean standardized log_2_ ratios in simple, complex and false positive deletions.**


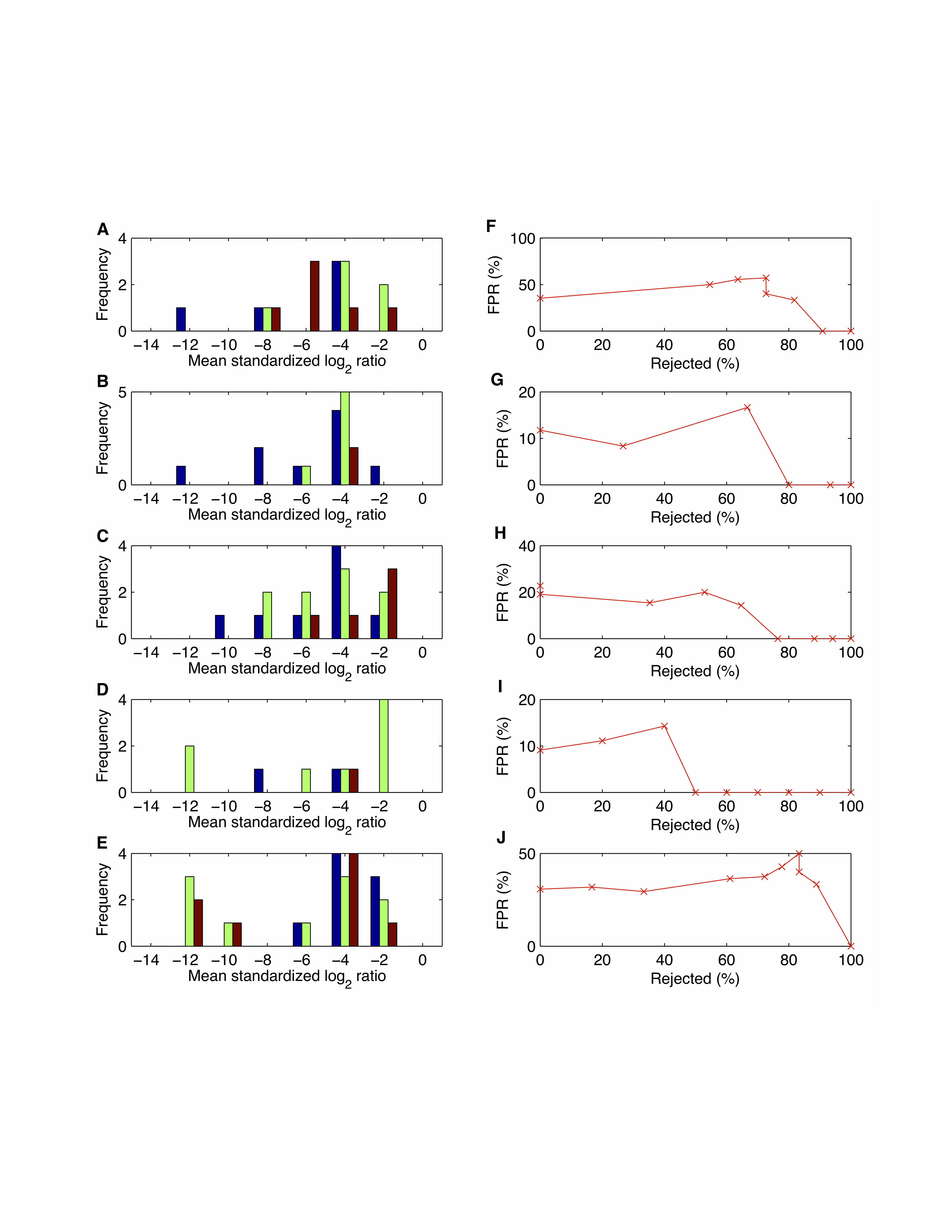


**A**-**E**: Distribution of the mean standardized log2 ratios in simple (blue), complex (green) and false positive (red) deletions in *AKR/J*, *CBA/J*, *DBA/2J*, *LP/J* and *C3H/HeJ*, respectively. **F**-**J**: We examined all thresholds for accepting a deletion between -1 and -14. For each threshold we plot the false positive rate (FPR) against the percentage of all verified deletions that are rejected. Results are shown in the same strain order as for plots A-E.
