# Supplementary material for: Elusive Copy Number Variation in the Mouse Genome
Source: PLoS One. 2010 Sep 21;5(9):e12839. doi: 10.1371/journal.pone.0012839 (PMC2943477; doi:10.1371/journal.pone.0012839)
Supplement: Figure S3 — Relationship between probe repeat content and hybridization. A: Box and whisker plots of the log2 ratios from probes in the 2.1M array A/J versus C57BL/6J normal dye experiment. Probes are grouped by repeat content. Probes either have no repetitive sequence at all, or they are found to have a minimum of 33% (this is due to the algorithm and default settings used by RepeatMasker). B: Chromosome 1 log2 ratio profile, with repetitive probes highlighted in green. Such probes constitute 11.1% of all probes. (0.06 MB DOC) [file pone.0012839.s003.doc]

**Figure S3 – Relationship between probe repeat content and hybridization.**


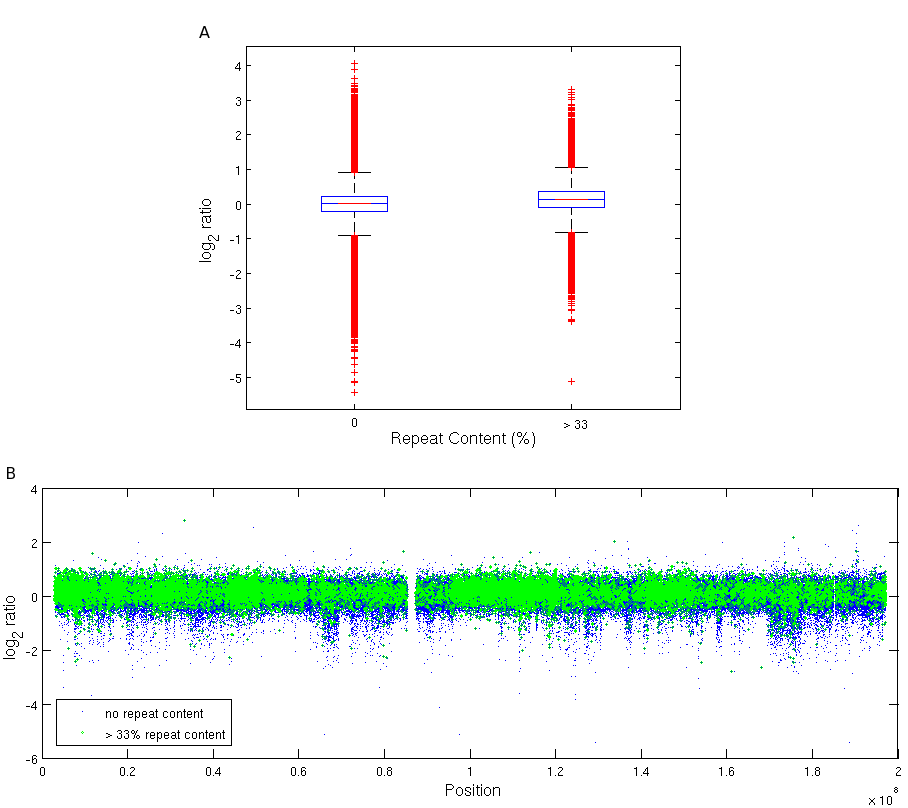


**A**: Box and whisker plots of the log_2_ ratios from probes in the 2.1M array *A/J* versus *C57BL/6J* normal dye experiment. Probes are grouped by repeat content. Probes either have no repetitive sequence at all, or they are found to have a minimum of 33% (this is due to the algorithm and default settings used by RepeatMasker). **B**: Chromosome 1 log_2_ ratio profile, with repetitive probes highlighted in green. Such probes constitute 11.1% of all probes.
