# Supplementary material for: Elusive Copy Number Variation in the Mouse Genome
Source: PLoS One. 2010 Sep 21;5(9):e12839. doi: 10.1371/journal.pone.0012839 (PMC2943477; doi:10.1371/journal.pone.0012839)
Supplement: Figure S4 — Distributions of observed and expected inter-CNV region distances. A: Histograms of the two distributions, with observed values in blue and expected in red. B: QQ-plot of the distributions. (0.30 MB DOC) [file pone.0012839.s004.doc]

**Figure S4 – Distributions of observed and expected inter-CNV region distances.**


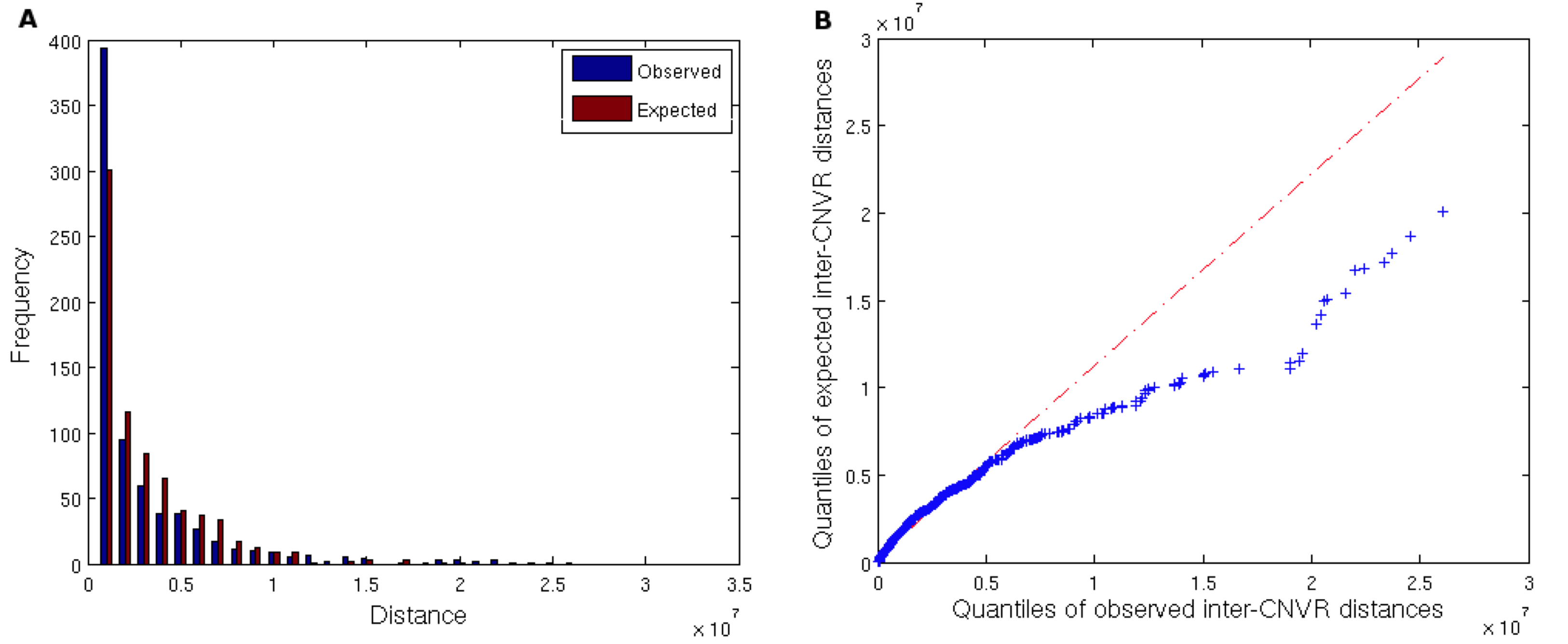


**A**: Histograms of the two distributions, with observed values in blue and expected in red. **B**: QQ-plot of the distributions.
