# Supplementary material for: Elusive Copy Number Variation in the Mouse Genome
Source: PLoS One. 2010 Sep 21;5(9):e12839. doi: 10.1371/journal.pone.0012839 (PMC2943477; doi:10.1371/journal.pone.0012839)
Supplement: Figure S5 — Schematic diagram of rotational permutation. 1: Start with CNV regions on a genome. The start and end of the genome are delimited by vertical green lines, the chromosomes by blue lines, and the CNV regions by red rectangles. 2: Wrap the genome into a circle. 3: Rotate the CNV regions by a random number of bases. 4: Unwrap the genome so that it is possible to measure the overlap with the biological attribute of interest. (0.15 MB DOC) [file pone.0012839.s005.doc]

**Figure S5 – Schematic diagram of rotational permutation.**

**
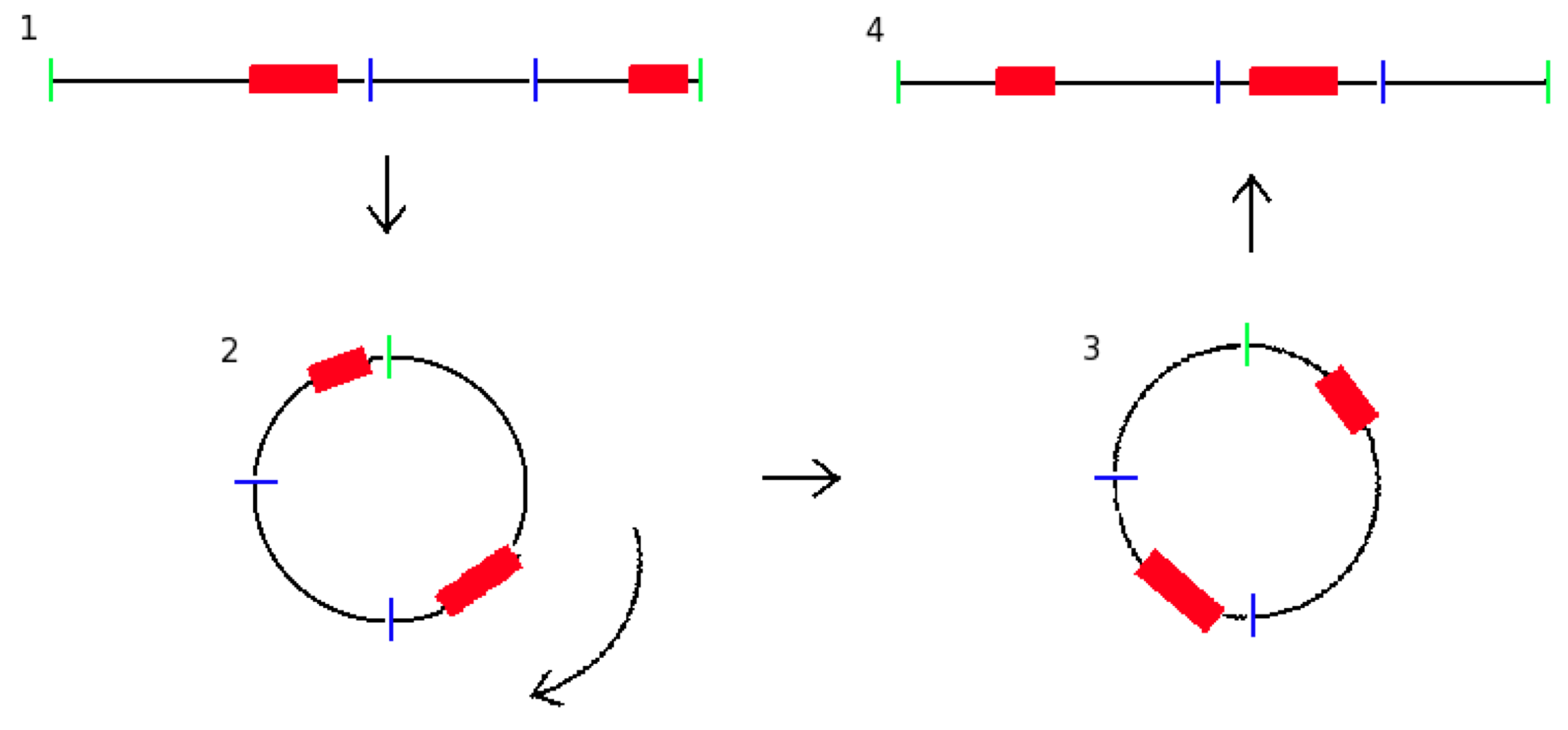
**

**1**: Start with CNV regions on a genome. The start and end of the genome are delimited by vertical green lines, the chromosomes by blue lines, and the CNV regions by red rectangles. **2**: Wrap the genome into a circle. **3**: Rotate the CNV regions by a random number of bases. **4**: Unwrap the genome so that it is possible to measure the overlap with the biological attribute of interest.
