# Supplementary material for: Elusive Copy Number Variation in the Mouse Genome
Source: PLoS One. 2010 Sep 21;5(9):e12839. doi: 10.1371/journal.pone.0012839 (PMC2943477; doi:10.1371/journal.pone.0012839)
Supplement: Materials S1 — (0.06 MB DOC) [file pone.0012839.s019.doc]

**(I) The Effect of Probe Repeat Content on Hybridization**

We wanted to explore the effect of probe repeat content on hybridization, where the repeat content of a probe is defined as the percentage of bases in the probe that are known mouse sequence repeats, identified using RepeatMasker [1].

We conjectured that a probe with repeat content would be subject to non-specific, or background, hybridization, and hence have a log_2_ ratio that would be difficult to interpret. In addition, NimbleGen offer array designs in which all probes have no repeat content; so there was motivation to investigate this area.

Figure S3 A shows box and whisker plots of the distributions of log_2_ ratios from probes classified by their repeat content. The most extreme log_2_ ratios are observed in probes that have no repeat content, whereas the median log_2_ ratios from probes that contain repeats are elevated in comparison to the median value for probes that do not. (Median log_2_ ratio for probes with no repeat content is 0.01, and for probes with repeat content is 0.14; P=0, Mann-Whitney U test, U =2.54 x 10^11^). Both of these observations are likely due to the increased non-specific (background) hybridization to probes with a high repeat content; for any probe that is in CNV, if it also has repetitive sequence then the CNV signal will be dampened as a result of non-specific hybridization, and background hybridization will likely increase the observed hybridization signal for any affected probes.

Thus, since approximately 11% of probes have a high repeat content, and because such probes appear to be distributed over the whole genome, (see Figure S3 B), it is likely that log_2_ ratios from these probes interfere with signal on the 2.1M array; they dampen the signal from CNV and cause false negative results (that is, by obscuring the signal from real CNVs). Furthermore, their signal due to CNV is somewhat intractable because the background hybridization is hard to predict. Therefore, we removed all such probes prior to our analysis.

**(II) Post-processing Steps to Improve Putative CNV Calls**

Based on initial putative CNV calls, we implemented two post-processing steps to remove those CNVs that were most likely to be false positives.

**Probe Densities of CNVs**

The distribution of probe densities in the putative CNVs was calculated and it was observed that some CNVs have a probe density which is much lower than the mean genome-wide density of 88 per 100 Kb. We visually inspected the CNVs and determined that they fell into two categories: either they were composed of sparsely but evenly spaced probes or they consisted of large regions with no probes, flanked by much shorter regions which had the normal density for the array. In the case of the former, it seemed unjustified to give these the same weighting as putative CNVs for which there was much more evidence; however, by the same token, it seemed unnecessary to remove putative CNVs which had a probe density that, although low for the 2.1M array, was comparable to that achieved on the lower density aCGH experiments that we had conducted (S. Methods), and that had been conducted elsewhere [2,3,4]. In the case of the latter, there was no evidence for the CNV in the majority of the region called and, furthermore, such CNVs were artificially inflating the total quantity of predicted CNV in the genome. Therefore, we devised a scheme whereby we could automatically remove putative CNVs with very large inter-probe distances and/ or very sparsely but evenly spaced probes, without removing CNVs that had a better probe density than that observed in lower density arrays. In this way, we aimed to remove false positives without removing too many true positives, hence improving upon a more rudimentary step in which all CNVs with probe density below a certain threshold are eliminated.

Since the median probe spacing on our lower density arrays was approximately 5 Kb, it seemed sensible to remove CNVs with inter-probe distances > 5 Kb. However, this is too strict when CNVs containing such gaps are much longer than this. Therefore, we decided to remove only those putative CNVs with inter-probe distances > 5 Kb, *and* whose sum of such gaps constitutes more than a third of the overall CNV length. Choosing this threshold is a subjective decision, and here it was based on visual inspection of the sets of rejected and accepted CNVs when the threshold was varied.

**Keeping Dye Swap Replicated CNVs**

As explained in the main text, for each strain, NimbleGen conducted a normal dye and a dye swap replicate experiment. Therefore two sets of putative CNVs were detected for each strain (one from each of the experiments); we decided to include only those CNVs that were at least partially replicated in both sets.

**(III) Motivation for Rotational Permutation**

To assess the structure and function of CNVs in the mouse genome it was useful to merge overlapping CNVs from different strains into discrete, non-overlapping, CNV regions [5]. Merging deletions yields 600 CNV regions, and merging gains yields 183. Combining the two sets gives 755 non-overlapping regions, which range in length from 1,070 bp to 5.48 Mb (mean 149.7 Kb, median 42 Kb). Thus they are longer than those found by Cahan et al. [6] (the only other study conducted on an array with a comparable probe density); there, more than 50% of CNV regions were less than 10 Kb, where as in this study only 12.9% are as short.

**CNV Regions Cluster**

The CNV regions are distributed across all 19 autosomal chromosomes, spanning approximately 4.5% of the autosomal genome, with minimum, median and maximum inter-CNV region distances of 1,131 bp, 1.3 Mb and 26 Mb. To ascertain whether or not the regions cluster we modeled the distribution of inter-CNV region distances under the null hypothesis that they are not clustered, by randomly permuting the CNV regions (that is, by allocating each one a random genomic position, without overlaps). We then compared the real, *observed*, inter-CNV region distances to those obtained after permutation (the *expected* distances). Figure S4 A depicts the two resultant distributions. Figure S4 B shows their QQ-plot. The central parts of the distributions are similar to one another, but there are many more short inter-CNV region distances in the observed data than there are in the expected data. Furthermore, referring to the QQ-plot, the tail of the observed distribution is much larger than the tail of the expected one. Finally, a χ^2^ test rejects the null hypothesis that the two distributions are the same as one another (d.f. = 11 (the data were split into 30 bins, but after pooling there were 12), *P* < 0.0005); this was also the case for a further 999 permutations of the CNV regions.

These results suggest that CNV regions cluster more than they would do if they were independent and occurred at a constant average rate on the genome; certainly, random permutation does not generate such clusters. Since we wanted to use permutation testing to assess the significance of the overlap observed between CNV regions and biological attributes, we needed to use another method of permutation, other than random, which maintains the inter-CNV region clustering. We therefore implemented rotational permutation.

**Rotational Permutation Algorithm**

In this algorithm the genome is viewed as a circle of contiguous chromosomes, with the end of chromosome 1 abutting the start of chromosome 2, and so on until the end of chromosome 19 meets the start of chromosome 1 (Figure S5, steps 1 and 2). Then, to permute the CNV regions we choose a random number X~U[1,N], where N is the total number of bases in the mouse genome, and rotate the coordinates of each CNV region by X bases along the circular genome (Figure S5, step 3). Thus, this method assigns randomized coordinates for each CNV region (step 4), whilst maintaining the distance between neighboring CNV regions.

**References**

1. Smit AFA, Hubley R, Green P (1996) RepeatMasker Open-3.0.

2. Henrichsen CN, Vinckenbosch N, Zollner S, Chaignat E, Pradervand S, et al. (2009) Segmental copy number variation shapes tissue transcriptomes. Nat Genet 41: 424-429.

3. Graubert TA, Cahan P, Edwin D, Selzer RR, Richmond TA, et al. (2007) A high-resolution map of segmental DNA copy number variation in the mouse genome. PLoS Genet 3: e3.

4. Cutler G, Marshall LA, Chin N, Baribault H, Kassner PD (2007) Significant gene content variation characterizes the genomes of inbred mouse strains. Genome Res 17: 1743-1754.

5. Redon R, Ishikawa S, Fitch KR, Feuk L, Perry GH, et al. (2006) Global variation in copy number in the human genome. Nature 444: 444-454.

6. Cahan P, Li Y, Izumi M, Graubert TA (2009) The impact of copy number variation on local gene expression in mouse hematopoietic stem and progenitor cells. Nat Genet 41: 430-437.
