# Supplementary material for: Elusive Copy Number Variation in the Mouse Genome
Source: PLoS One. 2010 Sep 21;5(9):e12839. doi: 10.1371/journal.pone.0012839 (PMC2943477; doi:10.1371/journal.pone.0012839)
Supplement: Methods S1 — (0.03 MB DOC) [file pone.0012839.s020.doc]

**NimbleGen 385,000 Probe Array**

We conducted a comparative genomic hybridization experiment using a NimbleGen long-oligonucleotide array containing ~385,000 50-75mer probes (385K array). The probes were selected from a Build 36 tiling database using a 5,250 bp interval and a 500 bp window. They span all chromosomes and are evenly spaced. Furthermore, the probes have been selected based on melting temperature rather than length (that is, they are isothermal) to ensure uniform hybridization behavior. In our analyses, only data from the 364,497 probes on the autosomal chromosomes were included. After re-mapping the probes to Build 37 of the mouse genome there were 364,450 remaining. The median probe spacing is 5.8 Kb, with 90th and 99th percentile spacing of 6.2 Kb and 17.4 Kb, respectively.

**Mouse DNA Samples**

The mouse DNA samples were taken from the same mice as those used in the NimbleGen 2.1M aCGH experiment (see Materials and Methods). We ran two experiments for each strain; a normal and a dye swap hybridization using DNA samples from the same animal.

**Array Processing and CNV Detection**

Normalization of hybridization signals was performed by NimbleGen using standard protocols. As described in the main text (Materials and Methods) for the 2.1M aCGH experiment, probes were annotated for SNP content before calling CNVs using SW-ARRAY. We applied a post-processing step to remove CNVs with a low probe density (< 4 probes per 100 Kb). Finally, only those CNVs that were at least partially replicated in both the normal and dye swap experiments were reported for each strain. Table S11 gives the summary statistics of the CNVs detected in each strain.
